# Next generation diagnostics on cardiomyopathy

**DOI:** 10.1186/1755-8166-7-S1-I4

**Published:** 2014-01-21

**Authors:** Jean-Louis Blouin, Jeremy Bevillard, Periklis Makrythanasis, Michel Guipponi, Federico Santoni, Stylianos E Antonarakis, Siv Fokstuen

**Affiliations:** 1 Genetic Medicine, University Hospitals of Geneva, Switzerland; 2 Genetic Medicine and Development, University of Geneva School of Medicine, Switzerland

## 

Cardiomyopathies are common, seemingly monogenic autosomal dominant cardiac disorders known as the primary cause of sudden cardiac death in young adults. These diseases are characterized by a remarkable genetic heterogeneity, which makes it difficult to unravel the causative mutation in a diagnostic laboratory that is very laborious and expensive by Sanger sequencing.

To circumvent these limitations, we explored solutions of high throughput sequencing of targeted exomes with the aim to implement this approach in routine diagnostics. As a first test we designed a capture microarray with the total genomic length of 1 Mbp that includes all exons/splicing sites of 130 genes involved in cardiovascular mendelian disorders and analyzed simultaneously four samples by multiplexing patients with cardiomyopathies or Long-QT syndrome. Pathogenic mutations and variants of unknown significance were found thus resolving the genetic causes of the cardiopathy in three. In the fourth patient the mutation usually associated with hypertrophic cardiomyopathy was found with Long-QT. Further developments to next generation diagnostics are now in progress, and will be also discussed.

In conclusion, high throughput sequencing holds considerable promises for molecular diagnosis of highly heterogeneous disorders in clinical practice and allows a better understanding of the complexity of mendelian disorders.

